# Characterizing first and third person viewpoints and their alternation for embodied interaction in virtual reality

**DOI:** 10.1371/journal.pone.0190109

**Published:** 2017-12-27

**Authors:** Henrique Galvan Debarba, Sidney Bovet, Roy Salomon, Olaf Blanke, Bruno Herbelin, Ronan Boulic

**Affiliations:** 1 Immersive Interaction Group, Ecole Polytechnique Fédérale de Lausanne, Lausanne, Switzerland; 2 Laboratory of Cognitive Neuroscience, Brain Mind Institute, Ecole Polytechnique Fédérale de Lausanne, Geneva, Switzerland; 3 Center for Neuroprosthetics, Ecole Polytechnique Fédérale de Lausanne, Geneva, Switzerland; 4 Artanim Foundation, Geneva, Switzerland; 5 Gonda Brain Research Center, Bar Illan University, Ramat Gan, Israel; 6 Department of Neurology, University Hospital Geneva, Geneva, Switzerland; Universite de Bretagne Occidentale, FRANCE

## Abstract

Empirical research on the bodily self has shown that the body representation is malleable, and prone to manipulation when conflicting sensory stimuli are presented. Using Virtual Reality (VR) we assessed the effects of manipulating multisensory feedback (full body control and visuo-tactile congruence) and visual perspective (first and third person perspective) on the sense of embodying a virtual body that was exposed to a virtual threat. We also investigated how subjects behave when the possibility of alternating between first and third person perspective at will was presented. Our results support that illusory ownership of a virtual body can be achieved in both first and third person perspectives under congruent visuo-motor-tactile condition. However, subjective body ownership and reaction to threat were generally stronger for first person perspective and alternating condition than for third person perspective. This suggests that the possibility of alternating perspective is compatible with a strong sense of embodiment, which is meaningful for the design of new embodied VR experiences.

## Introduction

The experience of embodiment, or bodily self-consciousness—the pre-reflective sensation of being the subject of an experience—comes from the coherent multisensory integration taking place in the brain and relates to the notion of an egocentric first person perspective on the self [1–5]. One feels embodied due “to the ensemble of sensations that arise in conjunction with being inside, having, and controlling a body” [6] (p. 374). It is proposed that the *sense of embodiment* emerges from three central components [6, 7], namely (i) the sense of agency, i.e. feeling of motor control over the body; (ii) the sense of body ownership, i.e. feeling that a perceived body is one’s own body; and (iii) self-location, i.e. the experienced location of the self. Although we experience our body as a consistent and seemingly immutable representation of our self in space, experimental protocols have shown that the sense of embodiment is much more malleable than commonly assumed. Conflicting multimodal stimulation can temporarily change how one perceives properties of their own body (i.e. an altered bodily self-consciousness). Notably, it can lead to the illusion of owning a fake—either material or virtual—limb [8–14], body [15–17], and even another individuals’ body [18, 19].

In the rubber hand illusion [8, 9], the synchronous stroking of a visible rubber hand and the occluded real hand provides visuo-tactile congruence to the subject, while causing a visuo-proprioceptive conflict. That is, the subject sees the rubber hand being stroked at the same place and time as she feels the stroke in the real hand, but the position of the rubber hand is offset relative to the proprioceptive perception of the real hand. As the brain tries to make sense of the multisensory incongruence, the conflict is often solved favoring the visuo-tactile congruence, and the subject feels ownership over the fake limb, which is accompanied by the feeling that the real hand is now located closer to the rubber hand (i.e. the congruent visuo-tactile stimulation induces alterations to the proprioceptive mapping). Additionally, this illusion can also be induced when active or passive movements of the hand (i.e. visuo-motor or visuo-proprioceptive congruence) are used *in lieu* of the tactile stimulation [13, 20, 21]. Moreover, alterations to the bodily self are not limited to body parts. Research using cameras and virtual reality (VR) demonstrated that a whole alien body can be felt as ones’ own body—in a full body ownership illusion—when visual, tactile and proprioceptive information match [16, 18]. Such changes in the experience of body ownership are often accompanied by changes in physiological processing such as skin temperature [22, 23] and increase in galvanic skin responses when the alien body is threatened [24].

The perspective from which the body is seen is another important aspect of illusory body ownership. The ownership over a body through multisensory congruence has been achieved using both first person perspective (1PP) [16–18] and third person perspective (3PP) [15, 19, 25–27], and differences in the ability to achieve it in 3PP were described [28, 29]. In the experiment proposed by [15], a 3PP image of a body is presented to the subject through a Head Mounted Display (HMD). A visuo-tactile stimulation synchronously delivered to the back of the subject and to the image of the body was shown to increase the sense of body ownership and to drift self-location closer to the seen body. This was not the case when the stimulation was asynchronous (i.e. with temporal mismatch between felt and seen stroking). In a follow up study, Slater et al. [16] directly compared 1PP and 3PP, and have further suggested that perspective is not only relevant, but also has a greater effect size on the reported sense of body ownership than the synchronous visuo-tactile stimulation.

In this paper we use VR to assess the effect of congruent visuo-motor-tactile feedback (full body control and haptic feedback vs. pre-recorded movements, which we refer to as VMT and ¬VMT conditions) and perspective (1PP and 3PP conditions) to the sense of embodiment of a virtual body. We additionally investigate how subjects behave when the possibility of alternating perspective at will is presented (ALT condition), and how the reported sense of embodiment of the virtual body in this condition compares to 1PP and 3PP alone. The ALT condition is proposed in order to integrate the advantages of both 1PP and 3PP viewpoints of the virtual avatar in a seamless experience of the virtual environment. The experiment consists of a series of tasks (reaching to targets, walk a few meters forward, feel a passive haptics device) that the subject had to perform (VMT condition) or watch the virtual body performing (¬VMT condition), and end up by exposing the subject to a virtual pit threat. Therefore, this study adds a new dimension to the consistency of multisensory cues by allowing the motor control of the whole virtual body with a natural mapping, including global aspects such as walking in the virtual environment and tactile congruence of the feet with floor and the beams of a platform.

The first objective of this experiment is to assess the viability of embodiment in 3PP when rich multisensory congruence is provided (congruent visuo-motor-tactile or not, VMT/¬VMT), and how it contrasts with 1PP. From a VR standpoint, 3PP allows taking a new and potentially more informative point of view within a VR application, such as for training [30–32]. For instance, 3PP is often employed in non-immersive virtual environments such as video games to increase awareness of the environment and threats to the player, thus overcoming field of view limitations of 1PP. In VR, the use of 3PP viewpoints have been recommended to help setting the posture of a motion controlled virtual body [30], and to compensate for the compression of distance perception inherent to immersion systems such as large stereoscopic projections [32]. The problem is that 3PP is not the natural condition in which subjects experience their real bodies, and might consequently lower the sense of ownership over the virtual body. The question is therefore to know if these benefits of 3PP could be exploited without detrimental consequences on the ability to embody an avatar.

This experiment secondly explores how subjects behave when the possibility of alternating between points of view in VR applications is presented, and how this affects their subjective sense of embodiment of the virtual body. Combining the best of the two approaches, 1PP maximizing embodiment and 3PP providing awareness of the surrounding, would open new possibilities in the design of Virtual Reality interaction. For instance, a VR experience could be started and developed in 1PP, and at moments where an overview of a situation, and the physical relation of the avatar with the environment is required, a temporary transition to 3PP could be conducted. Our hypothesis is that the rich multisensory congruence as well as the possibility of switching perspective at will can mitigate the negative effect of 3PP viewpoint to the sense of body ownership.

## Materials and methods

### Equipment and software

An Oculus development kit 2 **HMD** was used to display a virtual scene (960 x 1080 pixels per eye, 100° field of view, 75 Hz). Head tracking was performed using its inertial sensors (low latency) and corrected for drift around the vertical axis using optical tracking.

A pair of Bose^®^ QuietComfort 15 **headphones** was used for environmental noise canceling and to provide unlocalized white noise, thus phonically isolating the user from the real environment. Using a microphone, the experimenter could talk to the subjects directly through the headphones and provide instructions throughout the experiment.

A Nintendo^®^ Wii **remote controller** was used to allow the subjects to trigger when they would like to switch the perspective in the alternating condition. The Wii remote was also used for the mental ball drop task (see Response Variables). Subjects held the controller in their right hand. For consistency, the virtual avatar also held a similar object with the right hand.

**Galvanic skin response (GSR)** was acquired using a g.GSRsensor connected to a g.USBamp amplifier (g^®^.tec) and recorded with the OpenViBE software [33].

A Phasespace Impulse X2 optical tracking system was used for **motion capture**. Our Phasespace system uses 14 cameras and 40 markers attached to a motion capture suit and to the HMD. A VRPN [34] server interfaced the capture system (updated at 240 Hz) to the rendering engine (75Hz). An in-house analytical inverse kinematics implementation was employed to reconstruct the posture of the subject [35], which reinforces co-location of end effectors (hands and feet) with the equivalent physical markers. Fingers were not animated and were kept in a neutral pose. The body reconstruction latency from capture to render was approximately 40 to 50*ms*. To account for body size variability, a calibration based on a standard posture (T-stance) was performed until head, trunk and lower/upper limbs of the virtual body were adjusted in scale and orientation to closely match the real body.

**Fig 1:**
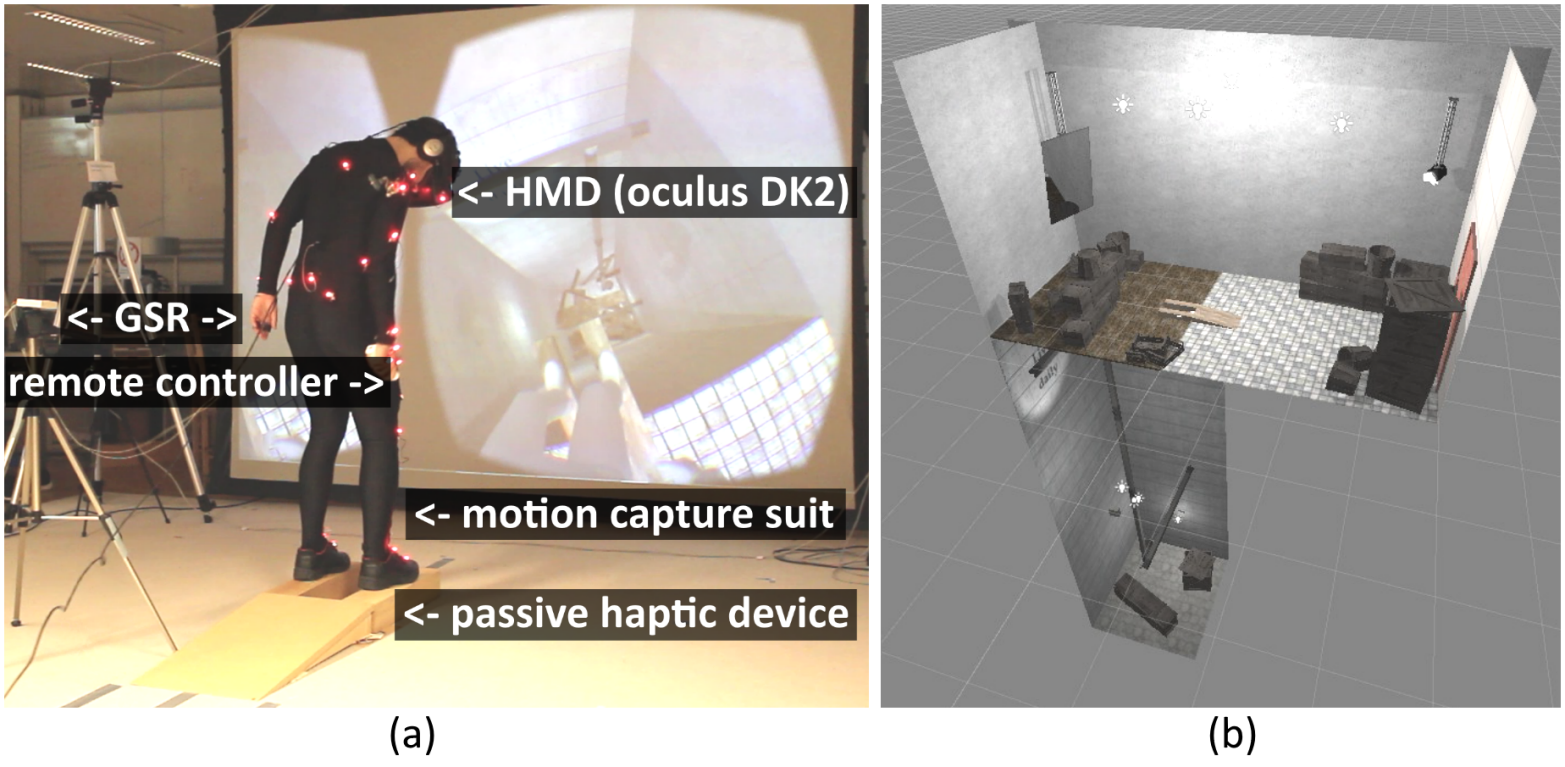
Experiment setup and scene overview. (a) The subject was fit with a motion capture suit, an Oculus DK2, GSR sensors and a Wii remote. Note that this picture was staged with one of the authors for illustrative purposes, during the experiment the lights were off and the projection display, which in the picture presents the point of view of the subject, was not used. (b) Presents an overview of the virtual scene.

A physical object and its virtual representation were used to convey congruent visuo-tactile stimulation when walking over the pit. This manipulation is known as **passive haptics**, when a seen virtual object has a physical equivalent, which is calibrated to spatially match, thus rendering accurate tactile sensations. This device is made of wood and its dimensions are 140*cm* × 40*cm* × 10*cm*. Fig 1a shows an overview of the experimental environment and the equipment the subject had to wear. Note that the picture shown in Fig 1a was staged for illustrative purposes. During the experiment the lights were off, and the

The **virtual environment** was developed using Unity 3D, and was inspired by the pit room proposed by Meehan et al. [36]. It featured a main room and a 10m deep virtual pit. The main room was 3.4 meter high and slightly smaller in surface than the captured space. A virtual mirror was placed over the pit, facing the virtual body. For each session, the pit was initially covered by a wooden floor. A wooden ramp was located in the center of the scene. During a session run, the floor covering the pit would eventually fall (at the command of the experimenter), revealing the pit to the subject and leaving the virtual body standing on the wooden ramp overseeing the pit. An overview of the virtual environment is presented in Fig 1b.

### Experiment design

The experiment had two manipulated variables and followed a mixed factorial design, with *multisensory congruence* as the between-subject variable and *perspective* as the within-subject variable. Multisensory congruence was treated as a between-subject variable for two reasons. First, the ¬VMT condition requires pre-recording movements from the VMT sessions, which is optimally achieved and randomized by using recording of the VMT group for the ¬VMT group. Second, in previous work studying the influence of perspective change and visuo-motor congruence on agency, body ownership and self-location [26], we have observed a limitation of the within subject design leading to a potential ceiling effect and under-evaluation of the perspective factor with respect to the congruence one. Conversely, within subject design was selected for the perspective factor as it limits the number of subjects, thus balancing the experimental time with the long preparation time needed for each subject.

Response variables were determined in order to assess components of the sense of embodiment, consisting of an embodiment questionnaire, the variation of GSR following a threat event, and a mental imagery task where the subject had to estimate the time an hypothetical ball would take to hit the ground (mental ball drop—MBD). The response variables are detailed later in the paper.

#### Multisensory congruence factor

Subjects were assigned to one of two equally sized groups. The first group performed the experiment in a congruent visuo-motor-tactile condition (VMT group), in which subjects could control the movement of the virtual body, had to perform a sequence of tasks and could interact with a passive haptic device that stands in between the virtual body and the bottom of the pit. The second group could not control the virtual body (¬VMT group), instead subjects were placed standing at the starting position and had to watch the virtual body moving as recorded from subjects in the VMT group. The lack of visuo-proprioceptive congruence with the virtual body is expected to negatively impact the senses of agency and ownership of the virtual body. As the motion recordings of the VMT group were necessary for the ¬VMT condition, we ran all subjects of that group before proceeding to the second group. The subjects in the ¬VMT group also wore the motion capture suit, thus allowing for similar GSR recording conditions.

Note that subjects in the ¬VMT group could still control the rotation of the virtual camera. This aspect was kept across groups because it is critical to prevent cybersickness, which is mainly attributed to the sensory mismatch of visual and vestibular systems [37], particularly when visual movement is present in the lack of its vestibular counterpart. In contrast, the position of the virtual camera had to be driven by the data recorded during the VMT sessions in order to grant consistent viewpoint location relative to the virtual body experienced by the VMT group. We assessed a smaller risk of sickness in this case as translations often results in smaller changes to the visual flow than rotations. As a result, subjects in the ¬VMT group experienced partially congruent sensorimotor feedback of the virtual camera.

#### Perspective factor

**Fig 2:**
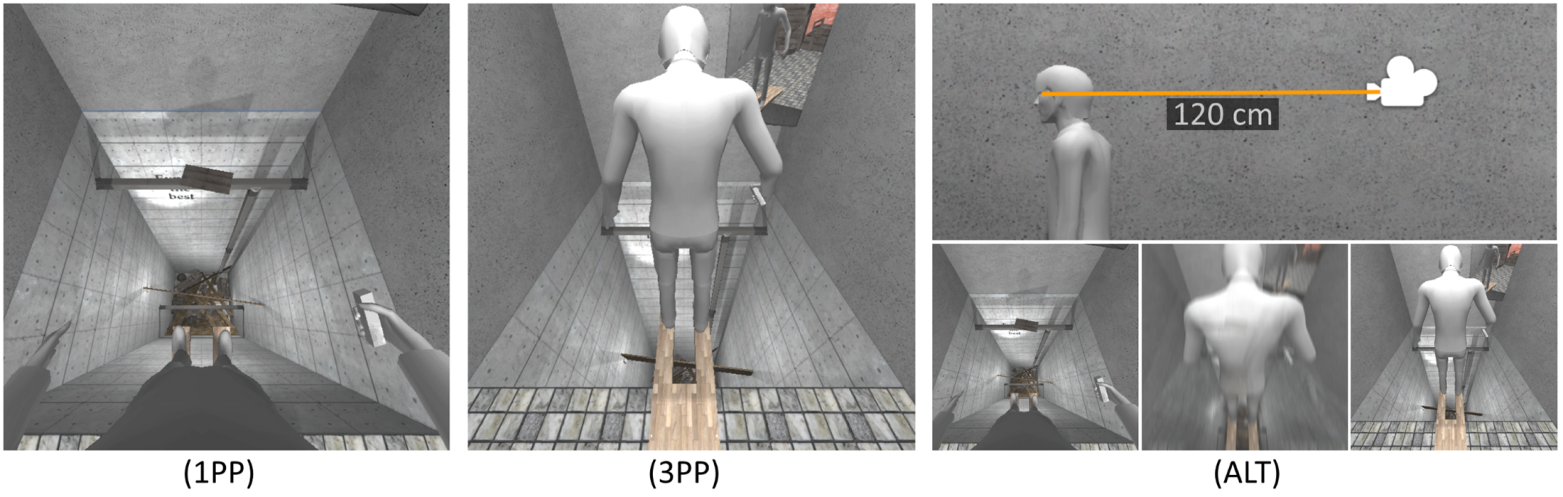
Perspective conditions. The subject could experience the scene in three different conditions: (1PP) first person perspective; (3PP) third person perspective; or (ALT) be free to alternate between 1PP and 3PP. When in the alternate condition, subject were asked to perform at least 3 perspective switches.

Each subject repeated the experimental session three times, once for each *perspective* condition: first person perspective (*1PP*), third person perspective (*3PP*), and a novel one in which the subject could *alternate* between 1PP and 3PP at will (*ALT*). This alternation of perspective required the implementation of a transition phase which was carefully designed to prevent cybersickness. Three different approaches were considered and tested. In the first one, camera followed for a second a parametric curve with accelerating and decelerating phases in order to avoid interpenetration with the virtual body. This was however not efficient as it required a long trajectory and continuous changes in the direction of movement and gave the false impression of real movement to the subject (some subjects would try to compensate and lose balance). The second alternative was teleportation which entirely avoids translation. However, teleportation is known to cause disorientation [38] and to affect subjects’ ability to immediately resume a task on the new point of view. Finally, we opted in favor of a very fast (200 ms) straight line translation of the camera (Fig 2ALT). The vision was slightly blurred during movement, making it unlikely that subjects could perceive interpenetration with the virtual body. This approach allowed subjects to quickly resume their action after a transition. None of our subjects reported feeling sickness with this transition.

The position of the camera in 1PP lies in between the eyes of the virtual body (Fig 21PP). The position of the 3PP camera was shifted 120cm toward the back of the scene and moving relatively to the head of the virtual body (Fig 23PP). This way, in 3PP condition, the virtual body is exposed to the threat of the pit while the virtual camera remains over a safe area (the floor, Fig 1b). In the *ALT* session, subjects could decide when to trigger the perspective switch by pressing a Wii remote button with the right thumb. They were instructed to perform this action at least three times during the session. The *perspective* presentation order was counterbalanced.

### Session overview

An experimental session was divided into 4 stages: REACH, WALK, WAIT and OBSERVE.

**Fig 3:**
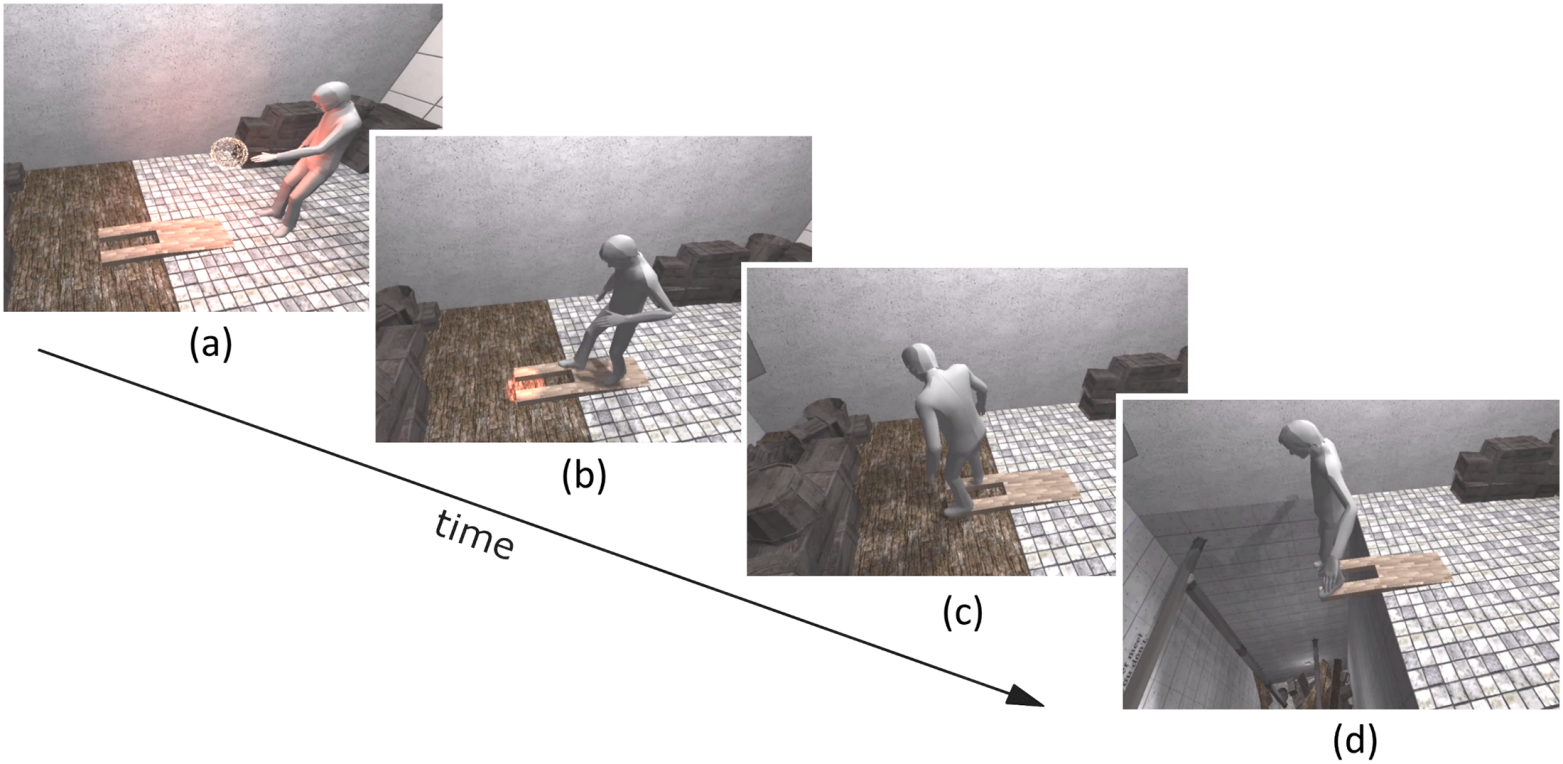
Overview of the session stages. (a) First the subject has to reach for targets that can appear either in the air or in the floor (REACH stage); (b) a final target invites the subject to walk to the wood platform (WALK); (c) once on the platform, the subject is asked to feel the edges with their feet (WAIT); (d) finally, the wooden floor beneath the platform collapses, revealing the pit to the subject (OBSERVE). Subjects in the ¬VMT group do not perform these task, instead they watch recordings from the VMT group. The session was followed by the mental ball drop (MBD) task and an embodiment questionnaire.

*REACH*: the subject had to reach 12 targets appearing around him/her (Fig 3a). There were six ground and six air-targets activated one after the other in a shuffled order. Between each target reach the subject had to place back both feet on a central target. The targets were placed such that they were at equal distance to the central target (ground targets), and to the chest of the participants (air targets).

*WALK*: a 13th target eventually lights up in front of the wooden ramp, inviting the subject to walk from the initial position to the edge of the ramp, i.e. on the passive haptic device (Fig 3b). The central target and the front of the ramp were separated by 2.1 meters.

*WAIT*: once the subjects arrive to the end of the ramp, they were orally instructed—through their headphones—to feel the edges of the ramp with their feet, sensing the passive haptic device while observing the virtual body simultaneously touching it (Fig 3c). During this event the experimenter would press a button, and the floor would fall down within 1 to 5 seconds (random), with a cracking sound (Fig 3d).

*OBSERVE*: the floor fall event marked the transition to the OBSERVE stage. In this stage the subjects were asked to read some words in the pit wall opposite to where the virtual body stands, so that they had to face the pit.

For the *¬VMT* group the virtual body was driven by the data recorded from the *VMT* group. No passive haptic device was used and the subject did not have to act to complete the session. The subject was told that the virtual body would move by itself, and that (s)he should pay attention to what the virtual body was doing. The camera position also moved according to the recording, but the camera rotation could still be controlled by the subject. We kept this level of control due to its critical role preventing cybersickness [37]. The session started with a short communication, and further communication followed to remind subjects to pay attention to the virtual body, and that they could not control it (in case they tried to). To assign the recordings to subjects in the ¬VMT group we have paired VMT and ¬VMT subjects, the pairing was random and assured that the subjects in both groups were assigned to the same perspective order, i.e. a ¬VMT subject that did the experiment in the 1PP, 3PP and ALT order used the recording of a VMT subject who did the experiment in that same order. We had to repeat some of the VMT group recordings due to a technical issues with the recording software used for the first 5 subjects.

### Response variables

#### Questionnaire

A **questionnaire** was designed to assess the senses of agency, body ownership, self-location and the effectiveness of the floor fall threat. It contains 10 questions, two related to each of the four measurements, and two controls. Questions were formulated based on related experimental protocols [7, 15, 39] and are presented in Table 1. The answers were given in a 7-point Likert scale, ranging from “Strongly DISAGREE” (-3) to “Strongly AGREE” (+3). We use the mean of the two related questions as the score of the four main response variables— *ownership*, *agency*, *self-location* and *threat* —, and the raw value for the two control question variables— *more bodies* and *turning virtual*. The questions were presented after each session in a random order.

**Table 1:** Embodiment questionnaire applied in the end of each session. Answers were given in a 7 point likert scale ranging from strongly disagree (-3) to strongly agree (3). The variable response corresponds to the mean score of the associated questions.

Variable name	Question: During the last session …
Agency	Q1 … it felt like I was in control of the body I was seeing
	Q2 … whenever I moved my body I expected the virtual body to move in the same way
Ownership	Q3 … I felt as if I was looking to my own body
	Q4 … it felt that the virtual body was my own body
Self-location	Q5 … it felt as if my body was located where I saw the virtual body to be
	Q6 … it seemed as if I were sensing the movement of my body in the location where the virtual body moved
Threat	Q7 … I felt as if the pit posed a threat to myself
	Q8 … it felt as if I could get hurt if the virtual body was to fall in the pit
More bodies	Q9 … it felt as if I had more than one body
Turning virtual	Q10 … it felt as if my real body was turning virtual

#### Galvanic skin response

GSR was recorded to assess physiological responses to the threat (floor fall event). We expect a GSR increase due to the threat, and the magnitude of this increase to correlate with the sense of body ownership. This type of measurement has been shown to be valid in stressful virtual environments by Meehan et al. [36], being present in the GSR signal of a subject even after multiple exposures. The electrodes were placed on the index and ring fingers of the subject and the GSR was recorded at a sampling rate of 512 observations per second. Our GSR response variable is defined as the difference between the median GSR in the interval between 1 and 6 seconds following the floor fall event, minus the median GSR in the 5 seconds preceding this event. Median GSR was preferred because some subjects presented a response that could vary beyond the ≈ 6*μS* (microsiemens) recording window that our setup allowed. A sample GSR recording for a complete session is presented in S1 Fig.

#### Mental ball drop

MBD is a mental imagery task adapted from [19]. In this task, the subject estimates the time a ball would take to fall down from their hand to the floor. This measurement was performed at the end of each session, when the virtual body was standing on the wooden ramp at the top of the pit. The MBD is meant to detect whether the subject have similar time estimation in 1PP and 3PP. Consistently shorter times in 3PP could indicate weak sense of self-location, as the subject might be using the bottom of the pit in 1PP, and the floor under the camera in 3PP.

Before performing this task the screen turned black, and the measurement was then performed with the subjects unaware of their surrounding. Subjects were instructed to press and hold the trigger button of the Wii remote controller to release the virtual ball, and to release the trigger button when they estimated that the ball have reached the floor. Subjects were not instructed about which floor they should consider (lab floor, point-of-view floor or pit floor). The task was repeated five times for each session, and the median of these trials gives the MBD time estimation for a given subject and condition.

#### Time in 1PP (specific to ALT usage)

Regarding the behavior of subjects while in the ALT condition, we evaluate whether the session stage (REACH, WALK, WAIT and OBSERVE) and multisensory congruence have an effect on the choice of perspective. To summarize the choice of perspective in the ALT condition we compute the proportion of time spent in 1PP (*time in 1PP* variable) during each stage of the ALT session (REACH, WALK, WAIT and OBSERVE) for both VMT and ¬VMT conditions. We evaluate whether the session stage and multisensory congruence have an effect on the choice of perspective. Moreover, to better understand the influence of the *time in 1PP* to the sense of embodiment, we verify if this variable is correlated with *ownership*, *agency*, *self-location*, *threat*, *GSR* and *MBD*.

### Analysis

**Statistical analysis** was conducted using R. For the response variables *agency, ownership, self-location, threat, more bodies* and *turning virtual*, the analysis was carried using mixed design analysis of variance (ANOVA) with *perspective* (1PP vs. 3PP vs. ALT) as a within-subject factor, and *multisensory congruence* (VMT vs. ¬VMT) and *perspective order* (1PP-3PP-ALT vs. 1PP-ALT-3PP vs. 3PP-1PP-ALT vs. 3PP-ALT-1PP vs. ALT-1PP-3PP vs. ALT-3PP-1PP) as between-subject factors. We included *perspective order* as a factor to verify if the order in which 1PP, 3PP and ALT have been presented could have had a consistent effect in the questionnaire responses. For *GSR*, a similar analysis was carried, but excluding the *perspective order* factor. For *MBD*, only the VMT group was considered, and repeated measures one-way ANOVA was used with Perspective as the independent variable.

As ANOVA assumes that the residuals of the model fit belong to a normal distribution, we tested this assumption with the Shapiro-Wilk test. If residuals are deemed not normal, we transform the response with a Box-Cox transformation *y*^*λ*^, which does not alter the order of the response values (monotonic transformation).

We conducted post-hoc analysis with pairwise t-tests and Holm-Bonferroni correction for multiple comparisons if a significant main effect of *perspective* or the interaction between *perspective* and *multisensory congruence* was found. For the latter we select a subset of possible comparisons in order to limit the correction of the significance level. More specifically, we fix the value of one of the variables, and test for the combinations of the other, and *vice versa*. This yields a total of 9 comparisons. We do not perform any post-hoc for significant effects related to *perspective order*, and simply report that a statistically significant effect has been found.

### Procedure

After reading the information sheet and completing the informed consent form, subjects were asked to fill in a characterization form with questions about their background (other experiments, experience with HMDs …) and physical characteristics (height, weight and age). Then the experimenter played a video demonstrating the stages of a session (Video S2 Video) and subjects were asked to wear the motion capture suit. Subjects in the *VMT* group had to undergo the motion capture calibration at this point. A brief training on how the mental ball drop (MBD) task should be performed followed, using the laboratory floor as a reference. Finally, the experimenter helped the subject fit the HMD and the noise canceling headphones, and tested the verbal communication through microphone. The GSR electrodes were placed in the left hand and the wii remote in the right hand. The subject then went through an experimental session. After the session was complete, the image on the HMD went black, and instructions of the MBD task appeared. The task was repeated 5 times, and then the experimenter removed the HMD and the headphones and asked the subject to fill in the embodiment questionnaire (Table 1). The session procedure was repeated three times, once per *perspective* condition. After the experiment subjects filled-in a post experiment questionnaire about their perspective of preference for different stages of the session. The questionnaire also asked whether they considered the floor of the laboratory or the floor of the virtual environment during the MBD task.

A total of 48 subjects participated on the experiment (8 females, age between 19—30, mean 22.6). All subjects had normal or corrected to normal vision, normal physical and psychological condition and did not suffer from acrophobia. For technical reasons and for optimal use of the motion capture system, we limited recruitment to subjects with height from 165 to 190 cm, and body mass index in the range from 18 to 27. Only 4 subjects reported having participated in an experiment using VR in the past, while 17 reported having tried a HMD in the past, one of which with weekly frequency.

This experiment was approved by the Commission cantonale d’éthique de la recherche sur l’être humain in Vaud (CERVD—protocol 02/13), Switzerland. Subjects, recruited through online registration system, had to read and sign a written informed consent form to participate and were compensated with 20 CHF/hour for their participation.

## Results

A summary of the results and details of the post-hoc statistical results are presented in S1 and S2 Tables. The data obtained with this experiment is available in S1 Data.

### Questionnaire

**Fig 4:**
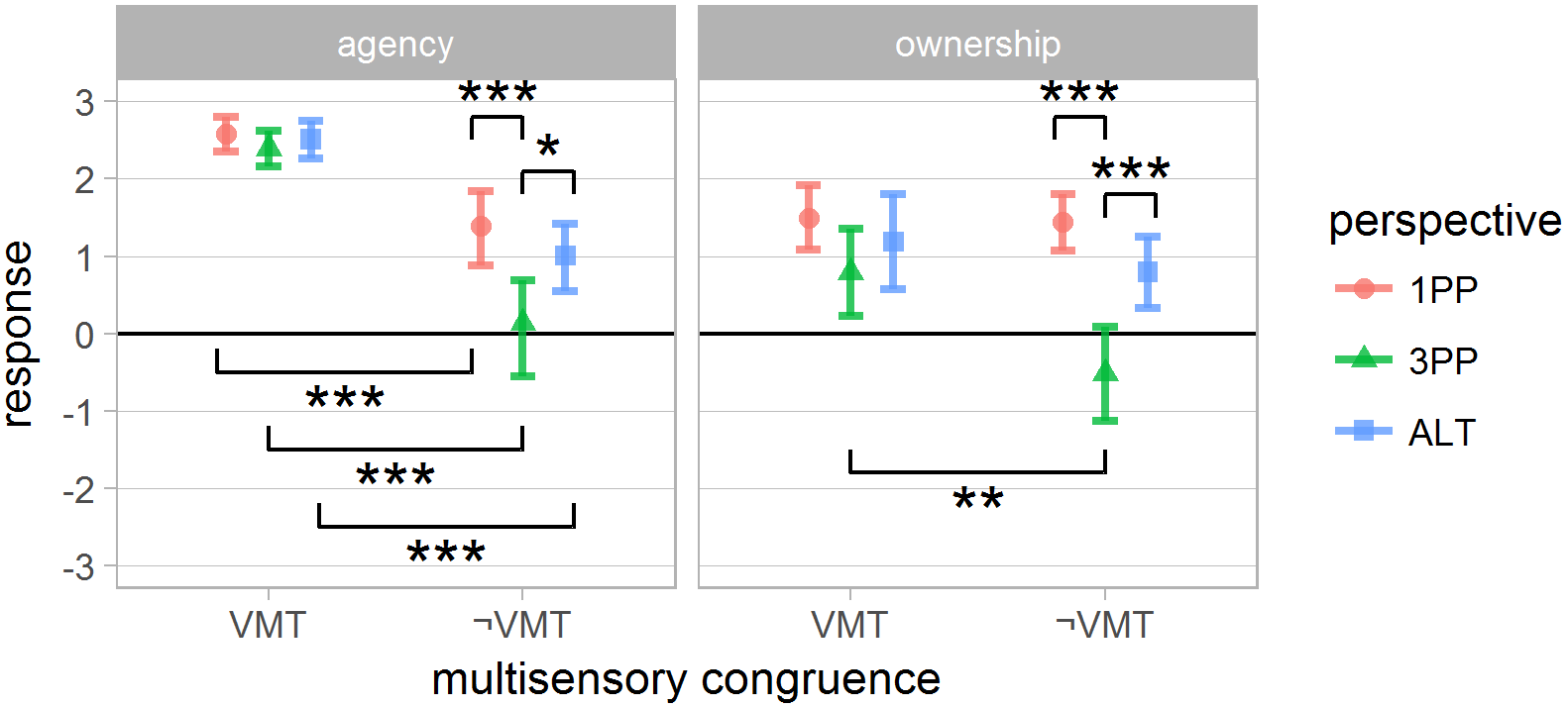
Questionnaire results: Senses of agency and body ownership for the interaction between *perspective* and *multisensory congruence*. Error bars represent the confidence interval of the mean (CI). “*”, “**” and “***” indicate *p* < .05, *p* < .01 and *p* < .001 respectively.

**Fig 5:**
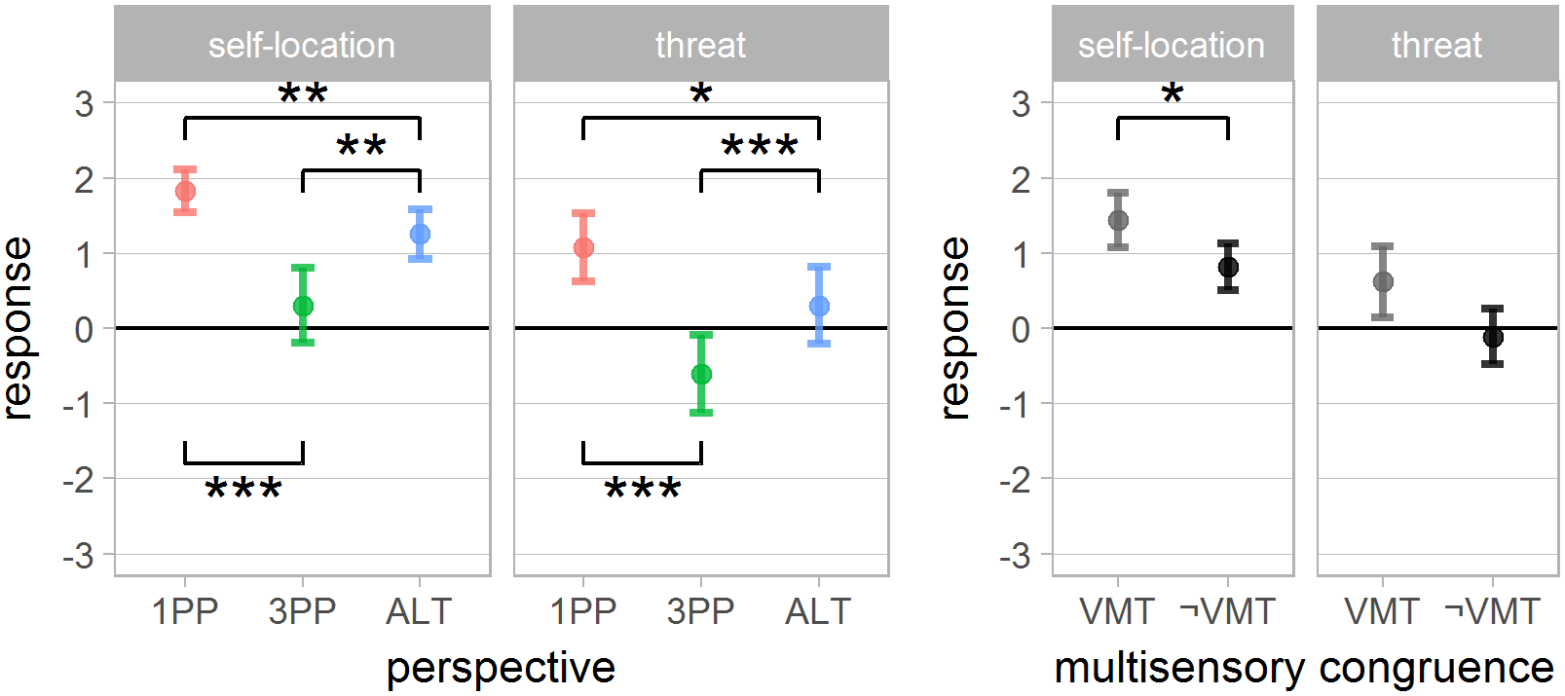
Questionnaire results: Self-location and threat responses for the main effect of *perspective* and *multisensory congruence*. Error bars represent the confidence interval of the mean (CI). “*”, “**” and “***” indicate *p* < .05, *p* < .01 and *p* < .001 respectively.

A summary of questionnaire results are presented in Figs 4 and 5.

*Agency:* agency response analysis yielded a significant effect of *multisensory congruence*, *perspective*, as well as their *interaction* (*F*_1,36_ = 98 *p* < .001, *F*_2,72_ = 8.7 *p* < .001 and *F*_2,72_ = 3.37 *p* < .05 respectively). The post-hoc of the interaction indicates a significant effect of *multisensory congruence* for all *perspective* conditions (VMT > ¬VMT). The sense of agency was significantly lower for 3PP when *multisensory congruence* was not present (1PP¬VMT and ALT¬VMT > 3PP¬VMT).

*Ownership:* a significant main effect of *multisensory congruence*, *perspective* and their interaction was found (*F*_1,36_ = 4.5 *p* < .042, *F*_2,72_ = 22.8 *p* < .0001 and *F*_2,72_ = 5.2 *p* < .008 respectively). Post-hoc of the interaction indicates that the response score in 3PP¬VMT was significantly lower than 1PP¬VMT, ALT¬VMT and 3PPVMT. The average ownership response was always positive when *multisensory congruence* was present, with no significant difference between *perspective* conditions in this case. It suggests that the lack of *multisensory congruence* negatively affected body ownership only for 3PP.

*Self-location:* showed a significant effect of *multisensory congruence* (VMT > ¬VMT), *perspective* and an interaction between *perspective* and *presentation order* (*F*_1,36_ = 4.3 *p* < .046, *F*_2,72_ = 33.8 *p* < .001 and *F*_10,72_ = 3.1 *p* < .003 respectively). Post-hoc analysis of the perspective factor shows a significant difference between all three conditions: 1PP > 3PP and ALT, and ALT > 3PP. The interaction with *perspective order* suggests that the perspective presentation order had influence over the reported self-location. Specifically, subjects starting the experiment in 1PP or ALT gave lower self-location scores to 3PP, while subjects starting in 3PP gave similar scores to all *perspective* conditions (presented in S2 Fig).

*Threat:* was significantly affected by the *perspective* factor (*F*_2,72_ = 21.4 *p* < .001). Post-hoc shows a significant difference for all *perspective* comparisons (1PP > 3PP and ALT, and ALT > 3PP). Although Fig 5 may suggest a consistent decrease of Threat score in the ¬VMT condition, the statistical test failed to reject the equality (*F*_1,36_ = 3.4, *p* > .075).

*More bodies:* a significant effect of *perspective* and its interaction with *multisensory congruence* was found (*F*_2,72_ = 4.3 *p* < .017 and *F*_2,72_ = 6.8 *p* < .003 respectively). Post hoc analysis of the interactions has shown statistically significant difference with 3PPVMT and 1PP¬VMT > 1PPVMT.

*Turning virtual:* a significant effect of *perspective* was found (*F*_2,72_ = 16.4 *p* < .001). Post hoc analysis shows that 1PP and ALT > 3PP.

### Galvanic skin response

**Fig 6:**
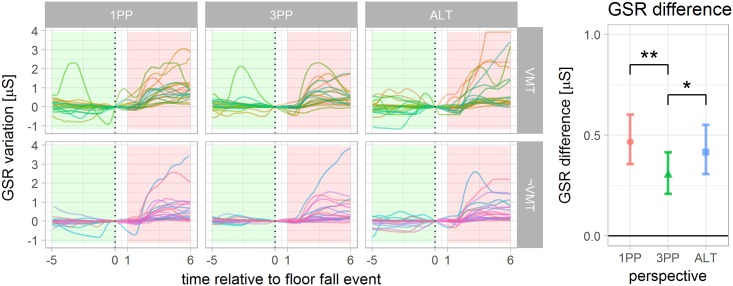
GSR variation time locked to the floor fall event (response in microsiemens). (left) The green and red shaded areas highlight the time interval used to compute the median GSR preceding (5 to 0 seconds before) and following (1 to 6 seconds after) the floor fall event for each subject. Each line color represents the GSR recording of one subject. The threat caused a statistically significant increase in the GSR response for all 6 combinations of conditions. (right) The difference between the medians is used to indicate the per subject GSR change linked to the threat. A significant difference between 1PP and 3PP was observed.

Eight subjects were excluded from the GSR analysis due to missing data or to failing connectors for at least one of the 3 sessions of the experiment. The recordings in the moments that precedes and follows the threat are presented in Fig 6. The threat event caused a significant increase of the median for all 6 possible combinations of conditions as compared by a pairwise Wilcoxon summed-rank test. When comparing the increase observed across the levels of *perspective* and *multisensory congruence*, ANOVA shows a significant effect of *perspective* (*F*_2,56_ = 4.21 *p* < .02). Post hoc shows a significantly stronger response in 1PP as compared to 3PP. The difference between ALT to 1PP and 3PP were not significant. The statistical test failed to reject the equality of VMT and ¬VMT (*F*_1,28_ = .59 *p* > .44), however, it is worth noting that GSR tends to present high inter-subject variability. GSR also presented a positive and statistically significant correlation with the Threat question (*r*_118_ = .34 *p* < .001), but not with Agency, Ownership or Self-location (*r*_118_ = .07 *p* > .45, *r*_118_ = .10 *p* > .26 and *r*_118_ = .17 *p* > .05 respectively). This suggests that the GSR was effectively related to how threatened the subject felt, validating the threat event. On the other hand, this measurement is usually expected to correlate with the sense of body ownership [17], although other experiments have also reported the lack of correlation [40].

### Mental ball drop

We noticed a bias of overestimating MBD time in the 3PP¬VMT condition. We believe this might result from limited visibility of the bottom of the pit due to the lack of body control in this specific condition. Thus, only the time of subjects performing in the VMT group were considered. One subject was excluded due to incomplete MBD data. The ANOVA test failed to reject the similarity of MBD time across *perspective* levels (*F*_2,44_ = 2.1 *p* > .14), making it unlikely that subjects performed the task differently in 1PP, 3PP and ALT conditions.

### Time in 1PP (specific to ALT usage)

**Fig 7:**
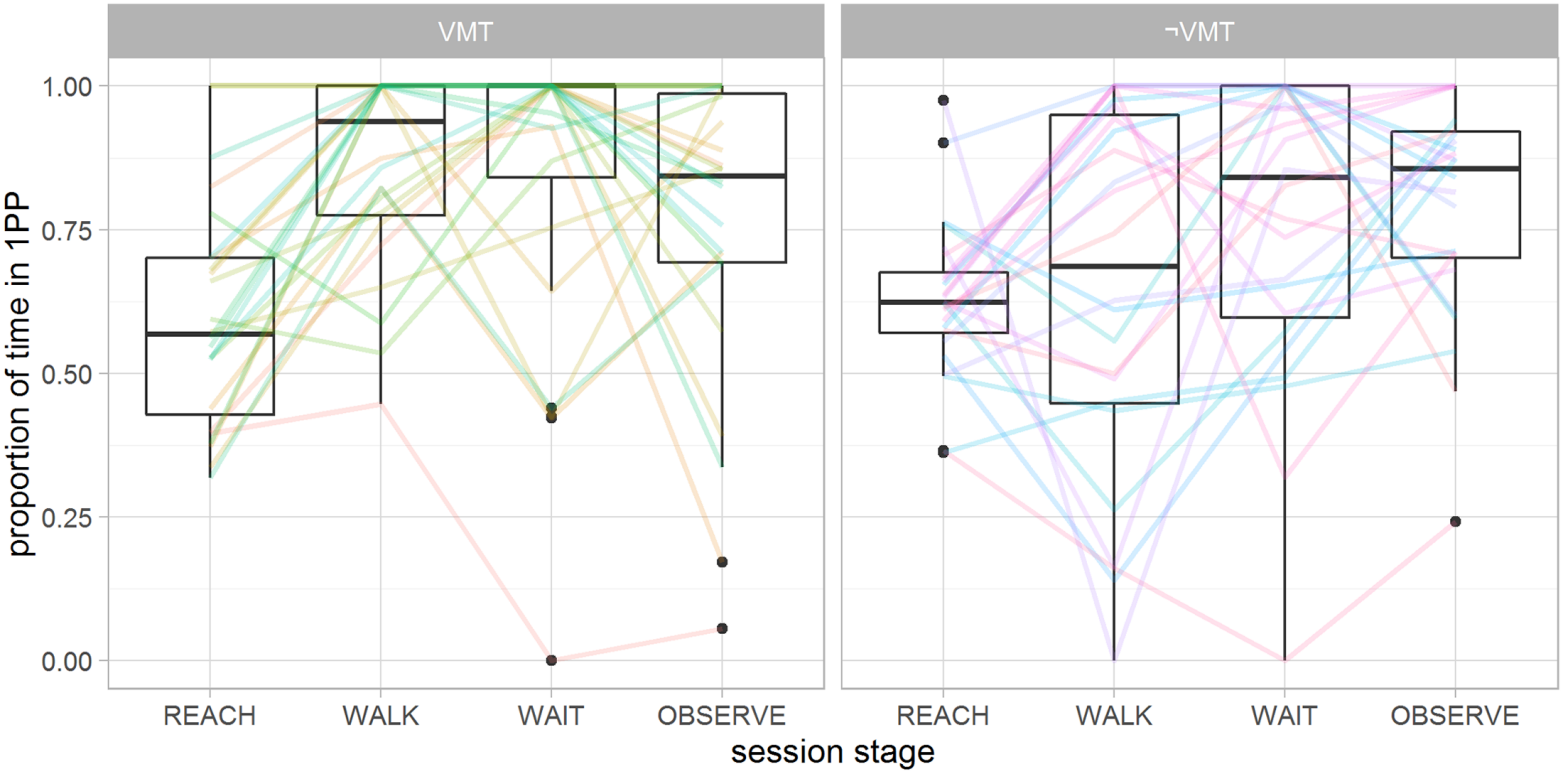
Breakdown of the proportion of time spent in 1PP for each stage of the ALT session for VMT and ¬VMT. Subjects tended to make a balanced use of perspectives in the REACH stage, while favoring 1PP for the following stages. Notably, overall perspective choice has shifted to 1PP once the reaching task was complete. 1PP seems to be preferred by the VMT group when they had to complete the walking task. This was not the case for the ¬VMT group, who had no practical incentive to change perspective at this stage of the session as the task is completed regardless of their actions. The WALK stage was the only one to present a statistically significant difference between the groups, as analyzed with pairwise t-tests (*t*_35_ = 2.88, *p* < .01).

Subjects performed 2 to 30 perspective switches during the ALT session, with mean±SD of 11 ± 5.6. Two subjects performed less perspective changes than instructed by the experimenter. The mean±SD proportion of time spent in 1PP was .68 ± .13. That is, nearly one third of the time in the ALT condition was spent in 3PP. Subjects tended to make use of perspective changes during the REACH stage, while favoring 1PP for the following stages. The breakdown of the proportion of time spent in 1PP during each stage is shown in Fig 7. The proportion of time in 1PP presents a significant positive correlation with the reported sense of self-location (*r*_46_ = .29 *p* < .05) and threat (*r*_46_ = .33 *p* < .022), but do not correlate with agency (*r*_46_ = −.04 *p* > .81) and ownership (*r*_46_ = .12 *p* > .4). The latter suggests that the possibility of alternating perspective had no consistent influence to the sense of ownership of the virtual body.

## Discussion

In our study we manipulated visual perspective (1PP, 3PP and ALT) and multisensory congruence (VMT and ¬VMT). Subjects could successfully perform all stages of all the sessions. We assessed the sense of embodiment with a questionnaire and the change in galvanic skin response due to a threat. Our threat was effective, and a clear and significant increase in GSR could be observed following the threatening event for all conditions. The results revealed several interesting findings. First, sense of body ownership measured in 3PP was similar to 1PP, but only when multisensory congruence was used, suggesting that visuo-motor-tactile congruence can mitigate the bodily discontinuities inherent to a 3PP view point in an ecologically valid VR experience scenario. Second, despite the lack of direct interaction with the virtual body in the ¬VMT group, subjects reported sense of agency and ownership of the body when in 1PP or ALT condition; this indicates that the match of having an intent and seeing the virtual body performing it may be sufficient to feel agency and body ownership in 1PP and ALT. Third, the ALT condition had similar response to 1PP, regardless of the multisensory congruence condition, indicating that it could be used in VR experiences as an alternative to having a constant point of view.

### Sense of embodiment in 3PP

The experimental manipulation of multisensory congruence had the expected effect on the 3PP condition. The 3PP:VMT group reported a significantly stronger sense of agency, body ownership and self-location than the 3PP:¬VMT group.

On the one hand, the sense of agency and small alterations to self-location of a body seen in 3PP through multisensory congruence are well supported by literature. Agency in humans represents an adaptive causal link, that seems to be constantly modelled by action and outcome contingencies developed by repetition [41]. One can feel agency over outcomes that are mediated by a device, such as a sound caused by pressing a button [42]. Thus, it is to be expected that agency over a controlled virtual body will be sustained independently of perspective, as reflected in our agency results (but see [21]). Moreover, alterations to the sense of self-location are also consistently reported [2, 15, 19, 25, 29, 43]. A currently supported hypothesis is that changes in self-location are produced by alterations of the peripersonal space (volume of space within body reach, which is associated to multisensory neurons reacting to visual/auditory and tactile stimulation [44, 45]), driven by the congruent tactile and/or motor stimulation, despite the incongruent point of view (3PP) [2]. Recent experimental protocols have found support to this hypothesis [29, 43]. In Noel et al. [43] the authors use an audio-tactile task to identify the point in space where a looming sound speeds up tactile processing. They replicate the protocol described in [15], showing that the peripersonal space drifts by a small amount towards the virtual body seen from a 3PP. Furthermore, novel results have shown that such modulation of self location and peripersonal space can be induced even when participants are unaware of the stimulation [46]. Overall, our self-location results indicate a positive response in 3PP, although significantly lower than in 1PP. Moreover, the results of the mental imagery task (MBD) suggests that subjects did not differentiate between 1PP and 3PP when estimating the time that an imaginary ball would take to hit the ground, regardless of the fact that the point of view was located 10 meters farther from the ground when in 1PP.

On the other hand, the sense of ownership of a virtual body in 3PP is a more subtle aspect of embodiment that requires further attention. First, our results are inline with previous experiments using visuo-tactile [15, 19] or visuo-motor [25, 26] congruence showing that ownership of a virtual body is possible in 3PP. They however contrast with other experiments where visuo-tactile synchrony and perspective were manipulated; Slater et al. [16] and Petkova et al. [17] show evidence of a strong influence of 1PP to the sense of ownership of a virtual body, more significant than the influence of visuo-tactile synchrony. Moreover, additional experiments by Maselli and Slater [28, 29] report subjects’ disagreement when asked about their experience of ownership of a body seen from 3PP. Here, our statistical analysis failed to reject the equivalency of body ownership between 3PP and 1PP in the VMT group in questionnaire responses. This contrasts with the clear evidence in the ¬VMT group that 1PP is a decisive factor for embodiment (as in [16, 17, 28, 29, 47]). Together, our questionnaire results suggest that most of the influence on the sense of body ownership were mitigated by the multisensory congruence in place. This might be explained by the new dimensions to the consistency of multisensory cues that our study provides; allowing motor control of the whole virtual body with a natural mapping and including global aspects such as walking in the virtual environment. In addition, our study adds an effective bodily involvement through the threat of falling, which is supported by a correlation between GSR and threat questionnaire scores. Interestingly, we do not find a correlation between GSR and body ownership score (which would be in line with [17]), yet we find a weaker response to threat—both GSR and questionnaire—in 3PP than in 1PP or ALT. We interpret this as an evidence that perspective had an impact on the subjective feeling of body ownership.

### 1PP and multisensory congruence

In addition to the expected results on 3PP, it is worth noticing that, in 1PP, the effect of multisensory congruence was verified for agency and self-location, but not for body ownership, thus suggesting a strong effect of perspective to the sense of body ownership only when no other congruent sensorial clues were present. This could be an appealing advantage for 1PP, as it suggests that observing the virtual body from a natural point of view while only controlling camera orientation is sufficient for the subject to self-identify with the avatar body, independently of proprioceptive and tactile congruence. Moreover, even though the responses to the agency questions were significantly inferior for ¬VMT, its absolute value are still positive, unveiling a degree of agreement with the sense of agency question statements. It could be hypothesized that subjects associated the control of the camera with the control of the head of the avatar, thus leading to a feeling of partially controlling the body.

These results find support on the recent work of Kokkinara et al. [48]. In their study, seated subjects developed the feeling of agency and ownership of a walking virtual body. But only when the externally controlled virtual body was experienced from a 1PP. The authors make the argument that, in line with the more subjective account of agency proposed by Synofzik et al. [49], the intention to walk may have been produced during observation, driving the self-attributing that they report. With the exception that the tasks in our experiment had higher complexity, our ¬VMT condition closely replicates their experimental paradigm, with compatible agency and body ownership results, and thus supporting their view.

### Alternating perspective

The ability to choose the point of view resulted in embodiment responses that were similar to 1PP, regardless of the multisensory congruence condition. This is most probably related to the larger amount of time spent on average in 1PP than in 3PP (Fig 7). Still, our results suggest that the relation with a virtual body experienced from 1PP can be sustained despite the periodic alternation to a 3PP point of view. Therefore, we observe that the ALT condition is a viable alternative for VR applications to maximize the sense of embodiment, without compromising the contextual information that 3PP can provide nor the more consistent bound to the virtual body that 1PP seems able to promote. We also highlight that more subjects preferred the ALT condition, and that they had the perception of performing faster in that condition, even though we found no clear effect of perspective in our performance measure (Table 2, a short analysis of the time to reach targets is available in S3 Fig). Moreover, the post experiment comparative questionnaire shows that subjects generally perceive the 3PP as safer than 1PP (Table 2). It is worth noting that none of the subjects reported feeling sick due to the perspective switch, although no formal testing has been conducted in this matter.

**Table 2:** Post-experiment responses for the VMT group. Values represent the total count of responses in favor of each perspective condition. Most subjects preferred to use 1PP, and felt safer in 3PP. When asked about conditions, subjects thought ALT to be more efficient in the reaching task. ALT was also preferred by more subjects than the other conditions.

Which point of view …	1PP	3PP	
… makes you feel safer when the floor falls?	3	**21**	
… do you prefer to use when the floor falls?	**19**	5	
… do you prefer to use to walk forward?	**22**	2	
… do you prefer to use to reach the targets?	**19**	5	
Which condition …	1PP	3PP	ALT
… do you prefer to perform the reaching task?	2	2	**19**
… is more efficient to reach the targets?	8	5	**10**

## Conclusion

In this paper we presented an experiment using Virtual Reality to explore the influence of perspective taking and multisensory congruence on the embodiment of a virtual body. We show that the multimodal correlation with the whole body movement and its physical contacts with the environment plays a prominent role on the sense of body ownership of a virtual body located in the extra-personal space (3PP), but is less influent when the point of view coincides with the body (1PP). Thus, in the context of the scientific debates investigating the influence of perspective taking, motor and sensory correlation over the sense of body ownership, our result stands out by supporting the view that a 3PP is compatible with body ownership when sensorimotor contingencies are present.

Moreover, we proposed and explored how a new method alternating 1PP and 3PP could benefit from the particular advantages of each viewpoint. Subjective evaluations of embodiment for this condition were very similar to those of 1PP alone, suggesting that the interruption of the point of view during the simulation is not significantly detrimental to the sense of body ownership of a virtual body. A potential application of alternated perspective could be in post traumatic stress disorder or phobia treatment, in which one can develop a strong sense of embodiment of the virtual body in 1PP, and then switch to 3PP when the body is exposed to a threat. This would allow the exposure to happen in a more reassuring manner, while still preserving a stronger bound to the virtual body, thus making the experience of self exposure flexible and the treatment more gradual.

In summary, our results contribute to the understanding of the interplay of the multiple components supporting embodiment and show that several factors (visuo-motor congruence, visuo-tactile congruence or perspective) can influence body ownership and embodiment depending on the tasks to perform and on the stimuli provided. Understanding the cognitive mechanisms of embodiment is a fundamental challenge for the development of VR interaction that needs to be investigated further. This study shows how an original idea for the design of interaction in VR can originate from and be supported by cognitive science knowledge, potentially leading to innovative interaction and navigation paradigms benefiting to several fields of application.

## Supporting information

S1 Video Overview of the experimental setup and conditions. (MP4)

S2 Video Session protocol. Video used to instruct the subject about the stages of a session.
(MP4)

S1 Fig Example of the GSR signal of a complete session. (PDF)

S2 Fig Reported sense of *self-location* at different levels of perspective and perspective order factors. (PDF)

S3 Fig Performance comparison of the reaching task (VMT group only). (PDF)

S1 Table Summary of results: Mean and confidence interval per experimental condition. (PDF)

S2 Table Summary of statistical tests results and their respective effect size estimations. (PDF)

S1 Data Data sets obtained with this experiment. (ZIP)
