# Supplementary material for: Characterizing first and third person viewpoints and their alternation for embodied interaction in virtual reality
Source: PLoS One. 2017 Dec 27;12(12):e0190109. doi: 10.1371/journal.pone.0190109 (PMC5744958; doi:10.1371/journal.pone.0190109)
Supplement: S1 Fig — (PDF) [file pone.0190109.s003.pdf]

Supporting Information - S1 Fig.

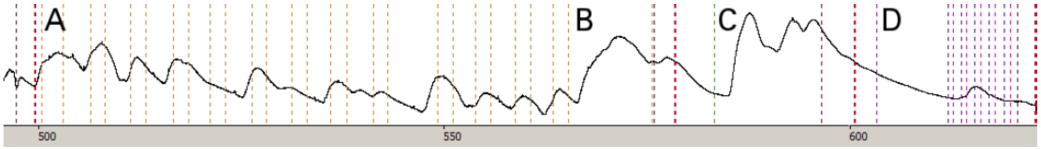

Example of the GSR signal of a complete session. The vertical lines indicate events transmitted by Unity to OpenVIBE. The REACH stage (A to B) usually takes most of the session time. For this specific signal one can observe increase of arousal when approaching the region of the threat (B), as well as after the onset of the threat (C). (D) marks the end of this session and the start of the MBD task.
