# Supplementary material for: Characterizing first and third person viewpoints and their alternation for embodied interaction in virtual reality
Source: PLoS One. 2017 Dec 27;12(12):e0190109. doi: 10.1371/journal.pone.0190109 (PMC5744958; doi:10.1371/journal.pone.0190109)
Supplement: S2 Fig — (PDF) [file pone.0190109.s004.pdf]

Supporting Information - S2 Fig.

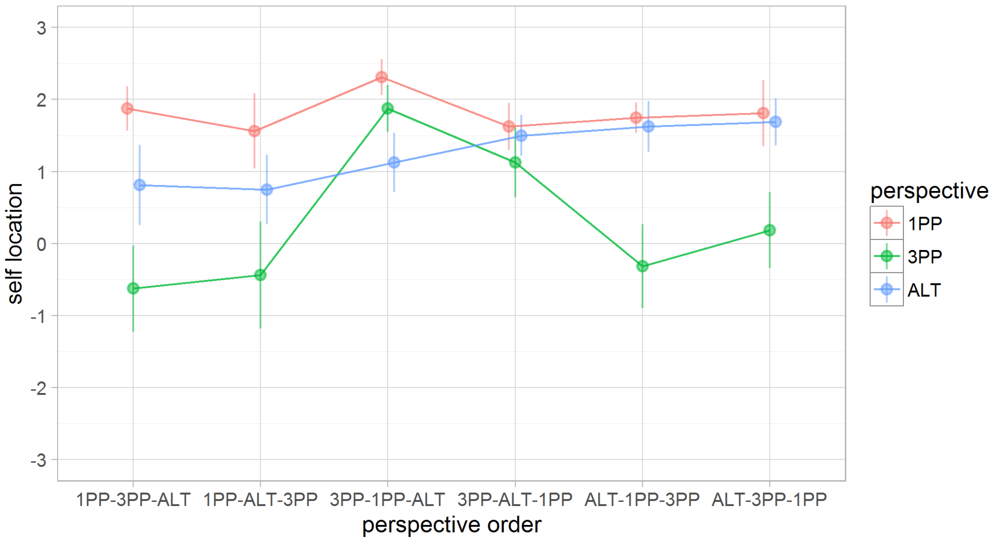

Reported sense of *self-location* at different levels of perspective and perspective order factors. Perspective order seems to influence reported sense of self-location for the 3PP condition. e.g. when answering self-location questions for 3PP after 1PP or ALT subjects tended to provide lower responses, which suggests a comparison bias.
