# Supplementary material for: Characterizing first and third person viewpoints and their alternation for embodied interaction in virtual reality
Source: PLoS One. 2017 Dec 27;12(12):e0190109. doi: 10.1371/journal.pone.0190109 (PMC5744958; doi:10.1371/journal.pone.0190109)
Supplement: S3 Fig — (PDF) [file pone.0190109.s005.pdf]

Supporting Information - S3 Fig.

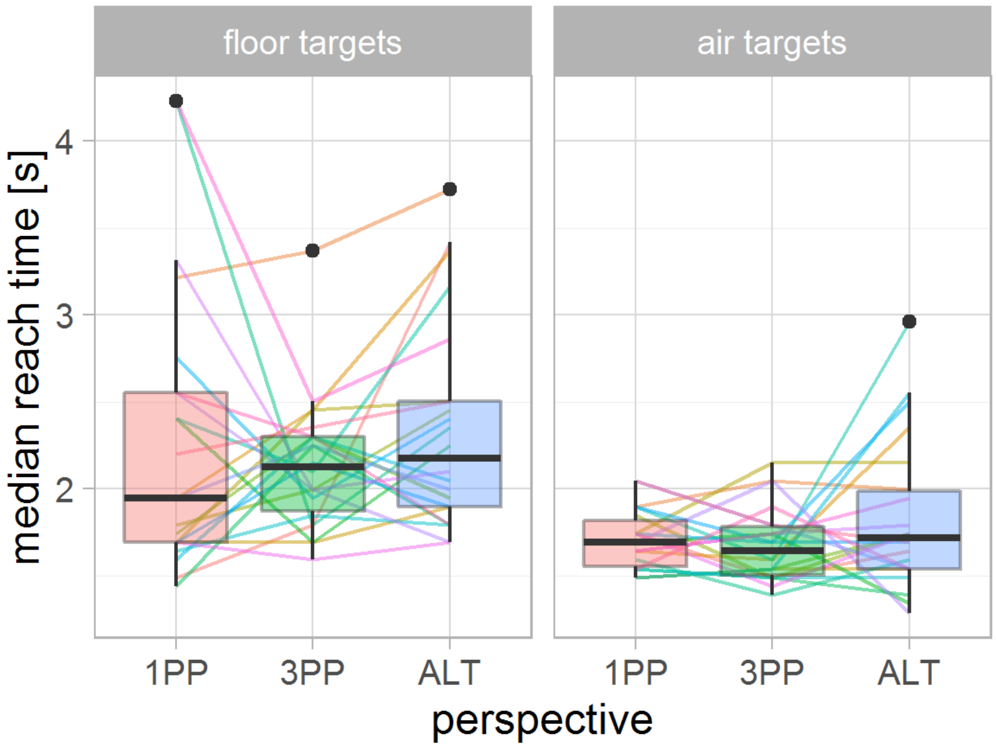

Performance comparison of the reaching task (VMT group only). The reaching performance was assessed with a two-way multiple comparisons ANOVA, with *Target position* (Ground vs. Air) and *Perspective* (1PP vs. 3PP vs. ALT) as the within subject variables. *Perspective* had no statistically significant effect on the time to reach targets ($F_{2,42} = 1.59$, $p > .21$), suggesting a similar performance across the three perspective conditions. The median time to reach across the reaching trials in a session was used as the performance measure.
