# Supplementary material for: Characterizing first and third person viewpoints and their alternation for embodied interaction in virtual reality
Source: PLoS One. 2017 Dec 27;12(12):e0190109. doi: 10.1371/journal.pone.0190109 (PMC5744958; doi:10.1371/journal.pone.0190109)
Supplement: S1 Table — (PDF) [file pone.0190109.s006.pdf]

Supporting Information - S1 Table

Summary of results: Mean and confidence interval per experimental condition

	VMT			¬VMT		
Response	1PP	3PP	ALT	1PP	3PP	ALT
Agency	$2.58^{+.22}_{-.23}$	$2.40^{+.23}_{-.24}$	$2.51^{+.23}_{-.25}$	$1.40^{+.45}_{-.51}$	$.14^{+.55}_{-.69}$	$1.01^{+.40}_{-.46}$
Ownership	$1.5 \pm .41$	$.79 \pm .56$	$1.19 \pm .61$	$1.44 \pm .35$	$-.52 \pm .61$	$.79 \pm .46$
Self-location	$2.19 \pm .36$	$.65 \pm .74$	$1.48 \pm .55$	$1.46 \pm .39$	$-.04 \pm .65$	$1.02 \pm .35$
Threat	$1.56 \pm .66$	$-.25 \pm .78$	$.54 \pm .87$	$.58 \pm .58$	$-.98 \pm .66$	$.06 \pm .54$
GSR	$.57^{+.21}_{-.16}$	$.35^{+.17}_{-.13}$	$.45^{+.22}_{-.17}$	$.38^{+.17}_{-.14}$	$.26^{+.16}_{-.13}$	$.39^{+.18}_{-.14}$

Mean score ± the confidence interval.
