# Supplementary material for: Characterizing first and third person viewpoints and their alternation for embodied interaction in virtual reality
Source: PLoS One. 2017 Dec 27;12(12):e0190109. doi: 10.1371/journal.pone.0190109 (PMC5744958; doi:10.1371/journal.pone.0190109)
Supplement: S2 Table — (PDF) [file pone.0190109.s007.pdf]

# Supporting Information - S2 Table

**Summary of statistical tests results and their respective effect size estimations.** Factors were examined using Mixed-design ANOVA and partial $\eta^2$ for effect size. Significant factors with more than two levels were examined using pairwise t-tests and Cohen's d for effect size.

| Variable | Multisensory congruence | Perspective | Interaction |
|---|---|---|---|
| Agency | $F_{1,36} = 98.0$ **p < .001** $\eta_p^2 = .73$ | $F_{2,72} = 8.71$ **p < .001** $\eta_p^2 = .20$ | $F_{2,72} = 3.37$ **p < .050** $\eta_p^2 = .09$ |
| Ownership | $F_{1,36} = 4.49$ **p < .042** $\eta_p^2 = .11$ | $F_{2,72} = 22.8$ **p < .001** $\eta_p^2 = .39$ | $F_{2,72} = 5.22$ **p < .008** $\eta_p^2 = .13$ |
| Self-location | $F_{1,36} = 4.31$, **p < .046** $\eta_p^2 = .11$ | $F_{2,72} = 33.8$, **p < .001** $\eta_p^2 = .48$ | $F_{2,72} = 0.30$ $p > .738$ $\eta_p^2 = .01$ |
| Threat | $F_{1,36} = 3.35$ $p > .075$ $\eta_p^2 = .09$ | $F_{2,72} = 21.4$ **p < .001** $\eta_p^2 = .37$ | $F_{2,72} = 0.47$ $p > .627$ $\eta_p^2 = .01$ |
| More bodies | $F_{1,36} = 3.84$ $p > .057$ $\eta_p^2 = .10$ | $F_{2,72} = 4.34$ **p < .017** $\eta_p^2 = .11$ | $F_{2,72} = 6.76$ **p < .003** $\eta_p^2 = .16$ |
| Turning Virtual | $F_{1,36} = 0.00$ $p > .946$ $\eta_p^2 = .00$ | $F_{2,72} = 16.4$ **p < .001** $\eta_p^2 = .31$ | $F_{2,72} = 0.74$ $p > .482$ $\eta_p^2 = .02$ |
| GSR | $F_{1,38} = 1.12$ $p > .295$ $\eta_p^2 = .03$ | $F_{2,76} = 6.39$ **p < .003** $\eta_p^2 = .14$ | $F_{2,76} = 0.65$ $p > .525$ $\eta_p^2 = .02$ |
| MBD | – | $F_{2,44} = 2.05$ $p > .141$ $\eta_p^2 = .09$ | – |

**Perspective** post hoc

| Variable | 1PP vs. 3PP | 1PP vs. ALT | 3PP vs. ALT |
|---|---|---|---|
| Self-location | $t_{72} = 6.66$ **p < .001** $d = 0.96$ | $t_{72} = 2.39$ **p < .020** $d = 0.35$ | $t_{72} = 4.27$ **p < .001** $d = 0.62$ |
| Threat | $t_{72} = 6.54$ **p < .001** $d = 0.94$ | $t_{72} = 2.99$ **p < .004** $d = 0.43$ | $t_{72} = 3.55$ **p < .002** $d = 0.51$ |
| Turning virtual | $t_{72} = 5.25$ **p < .001** $d = 0.76$ | $t_{72} = 0.65$ $p > .519$ $d = 0.01$ | $t_{72} = 4.61$ **p < .001** $d = 0.65$ |
| GSR | $t_{76} = 3.46$ **p < .003** $d = 0.50$ | $t_{76} = 0.97$ $p > .335$ $d = 0.14$ | $t_{76} = 2.49$ **p > .030** $d = 0.36$ |

**Interaction** post hoc

| | | VMT | |
|---|---|---|---|
| Variable | 1PP vs. 3PP | 1PP vs. ALT | 3PP vs. ALT |
| Agency | $t_{72} = 1.11$ $p > .819$ $d = 0.23$ | $t_{72} = 0.42$ $p > .994$ $d = 0.09$ | $t_{72} = 0.68$ $p > .994$ $d = 0.14$ |
| Ownership | $t_{72} = 2.50$ $p > .087$ $d = 0.52$ | $t_{72} = 1.10$ $p > .820$ $d = 0.23$ | $t_{72} = 1.40$ $p > .665$ $d = 0.29$ |
| More bodies | $t_{72} = 3.93$ **p < .002** $d = 0.80$ | $t_{72} = 2.50$ $p > .102$ $d = 0.51$ | $t_{72} = 1.43$ $p > .532$ $d = 0.29$ |

| | | ¬VMT | |
|---|---|---|---|
| Variable | 1PP vs. 3PP | 1PP vs. ALT | 3PP vs. ALT |
| Agency | $t_{72} = 4.72$ **p < .001** $d = 0.96$ | $t_{72} = 1.66$ $p > .409$ $d = 0.38$ | $t_{72} = 3.06$ **p < .016** $d = 0.63$ |
| Ownership | $t_{72} = 6.92$ **p < .001** $d = 1.41$ | $t_{72} = 2.28$ $p > .127$ $d = 0.47$ | $t_{72} = 4.64$ **p < .001** $d = 0.95$ |
| More bodies | $t_{72} = 0.98$ $p > .532$ $d = 0.20$ | $t_{72} = 1.52$ $p > .532$ $d = 0.31$ | $t_{72} = 2.50$ $p > .102$ $d = 0.51$ |

| | 1PP | 3PP | ALT |
|---|---|---|---|
| Variable | VMT vs. ¬VMT | VMT vs. ¬VMT | VMT vs. ¬VMT |
| Agency | $t_{97.39} = 5.51$ **p < .001** $d = 1.12$ | $t_{97.39} = 8.67$ **p < .001** $d = 1.77$ | $t_{97.39} = 6.59$ **p < .001** $d = 1.35$ |
| Ownership | $t_{82.97} = 0.17$ $p > .863$ $d = 0.04$ | $t_{82.97} = 3.63$ **p < .004** $d = 0.74$ | $t_{82.97} = 1.09$ $p > .820$ $d = 0.22$ |
| More bodies | $t_{99.14} = 3.10$ **p < .021** $d = 0.63$ | $t_{99.14} = 1.27$ $p > .532$ $d = 0.26$ | $t_{99.14} = 2.22$ $p > .142$ $d = 0.45$ |
