# Supplementary material for: Characterizing first and third person viewpoints and their alternation for embodied interaction in virtual reality
Source: PLoS One. 2017 Dec 27;12(12):e0190109. doi: 10.1371/journal.pone.0190109 (PMC5744958; doi:10.1371/journal.pone.0190109)
Supplement: S1 Data — (ZIP) [file pone.0190109.s008.zip › S1_data/data_book.pdf]

Databook

This package accompanies the submission “Characterizing First and Third Person Viewpoints and their Alternation for Embodied Interaction in Virtual Reality”. It contains 7 datasets related to the described experiment and presented results. All datasets are in “.csv” format, and can be easily loaded by statistical analysis tools (e.g. a dataset can be loaded in **r** using the command **read.csv(“filename.csv”)**).

A short description of the content of each dataset is presented below:

Embodiment_Q.csv

Sense of embodiment questionnaire data.

- **subject** - numerical ID of the subject
- **perspective** - the within subject independent variable *perspective* (1PP, 3PP or ALT)
- **mocap** - the between subject independent variable *multisensory congruence*, i.e. visuomotortactile congruence or not (VMT $\not{V}$MT)
- **p.order** - the between subject independent variable *perspective order* (1PP-3PP-ALT, 1PP-ALT-3PP, 3PP-1PP-ALT, 3PP-ALT-1PP, ALT-1PP-3PP and ALT-3PP-1PP)
- **Q1 to Q10** - the *embodiment questionnaire* responses per experimental session in a Likert scale from -3 to 3. The questions were:
  - “During the last session . . . ”
  - **Q1** “. . . it felt like I was in control of the body I was seeing”
  - **Q2** “. . . whenever I moved my body I expected the virtual body to move in the same way”
  - **Q3** “. . . I felt as if I was looking to my own body”
  - **Q4** “. . . it felt that the virtual body was my own body”
  - **Q5** “. . . it felt as if my body was located where I saw the virtual body to be”
  - **Q6** “. . . it seemed as if I were sensing the movement of my body in the location where the virtual body moved”
  - **Q7** “. . . I felt as if the pit posed a threat to myself”
  - **Q8** “. . . it felt as if I could get hurt if the virtual body was to fall in the pit”
  - **Q9** “. . . it felt as if I had more than one body”
  - **Q10** “. . . it felt as if my real body was turning virtual”
- **agency** - the *sense of agency* response variable, obtained as the mean of **Q1** and **Q2**
- **ownership** - the *sense of ownership* response variable, obtained as the mean of **Q3** and **Q4**
- **self.location** - the *sense of self-location* response variable, obtained as the mean of **Q5** and **Q6**
- **threat** - the *threat* response variable, obtained as the mean of **Q7** and **Q8**
- **more.bodies** - the *more bodies* response variable, obtained with the raw result of **Q9**
- **turning.virtual** - the *turning virtual* response variable, obtained with the raw result of **Q10**

GSR_Summary.csv

Summary results of the GSR physiological response to a thread (the floor fall event).

- **subject** - numerical ID of the subject
- **perspective** - the within subject independent variable *perspective* (1PP, 3PP or ALT)
- **mocap** - the between subject independent variable *multisensory congruence*, i.e. visuomotortactile congruence or not (VMT $\not{V}$MT)
- **GSR.median.before** - median GSR of the 5 seconds interval preceding the onset of the threat (in microsiemens)
- **GSR.median.after** - median GSR of the interval between 1 and 6 seconds following the onset of the threat (in microsiemens)

- **GSR.difference** - the *GSR* response variable, obtained by subtracting the **GSR.median.before** from the **GSR.median.after** (in microsiemens)

GSR_Recording_Cut.csv

This dataset contains the 11 seconds of Galvanic Skin Response (GSR) recordings used to assess the physiological response to a thread (the floor fall event).

- **subject** - numerical ID of the subject
- **perspective** - the within subject independent variable *perspective* (1PP, 3PP or ALT)
- **mocap** - the between subject independent variable *multisensory congruence*, i.e. visuomotortactile congruence or not (VMT $\not{V}$MT)
- **relative.time** - recording *time* relative to the onset of the floor falling event.
- **relative.gsr.signal** - the raw *gsr signal* minus the *gsr signal* at the first frame of the floor falling onset.
- **thread.stage** - stage of the floor falling event
  - *before* = 5 seconds interval prior to threat
  - *onset* = 1 second interval after start of floor fall event
  - *after* = 5 seconds interval after the end of the *onset* interval

MBD_Summary.csv

Dataset of the mental imagery task used in the experiment (mental ball drop - MBD)

- **subject** - numerical ID of the subject
- **perspective** - the within subject independent variable *perspective* (1PP, 3PP or ALT)
- **mocap** - the between subject independent variable *multisensory congruence*, i.e. visuomotortactile congruence or not (VMT $\not{V}$MT)
- **mbd.time** - median of 5 trials of the mental ball drop task.

Alternate_Use.csv

Summary of how and when subjects made use of the alternating point-of-view feature provided in the *ALT perspective condition.*

- **subject** - numerical ID of the subject
- **perspective** - only valid for alternating perspective condition (ALT)
- **mocap** - the between subject independent variable *multisensory congruence*, i.e. visuomotortactile congruence or not (VMT $\not{V}$MT)
- **REACH/WALK/WAIT/OBSERVE/SESSION.P.changes** - number of perspective alternations during the *REACH*, *WALK*, *WAIT* and *OBSERVE* stages, and during the whole *SESSION.*
- **REACH/WALK/WAIT/OBSERVE/SESSION.1PP.proportion** - proportion of time spent in first person perspective (1PP) during the REACH, WALK, WAIT and OBSERVE stages, and during the whole SESSION.

Reaching_Performance.csv

Reaching performance data of subjects. It is only valid for subjects in the *visuomotortactile congruence (VMT) group* .

- **subject** - numerical ID of the subject
- **perspective** - the within subject independent variable *perspective* (1PP, 3PP or ALT)
- **mocap** - only valid for visuomotortactile congruence condition (VMT)
- **floor.targets** - median of the time taken to reach targets that appeared in the floor

- **air.targets** - median of the time taken to reach targets that appeared in the air

Info_PreQ_PostQ.csv

General info about subjects and assigned conditions, pre-experiment characterization questionnaire, and post-experiment comparison questionnaire.

- **subject** - numerical ID of the subject
- **mocap** - assigned multisensory congruence condition
- **p.order** - assigned perspective order
- **playback.recording** - which VMT subject recording was used for playback a given VMT subject
- **complete.log.of.events** - whether the log of events of this subject was complete
- **valid.gsr** - whether the GSR signal recordings for the three sessions of this subject were valid
- **height** - the height of the subject
- **weight** - the weight of the subject
- **age** - the age of the subject
- **handedness** - the dominant hand of the subject
- **gender** - the gender of the subject
- **field** - field of study and/or interest of the subject
- **is.student** - whether the subject is a student
- Questions about the Virtual Reality background of subjects (1 = never, 2 = a few times, 3 = every month, 4 = every week and 5 = every day)
  - **VR.experiments** - "How often do you participate on experiments using Virtual Reality equipments?"
  - **use.HMD** - "How often do you use head mounted displays?"
  - **video.games** - "How often do you play video games?"
  - **motion.controllers** - "How often do use the Microsoft Kinect, Nintendo Wii or Playstation move?"
- Post questionnaire about perspective conditions:
  - "Which point of view ..." (1PP or 3PP)
  - **safer.when.floor.falls** - "... makes you feel safer when the floor falls?"
  - **prefer.when.floor.falls** "... do you prefer to use when the floor falls?"
  - **prefer.to.walk.forward** "... do you prefer to use to walk forward?"
  - **prefer.to.reach.tgts** "... do you prefer to use to reach the targets?"
  - "Which condition ..." (1PP, 3PP or ALT)
  - **prefer.condition.tgts** "... do you prefer to perform the reaching task?"
  - **efficient.condition.tgts** "... is more efficient to reach the targets?"
